# Effects of Lithium and Valproic Acid on Gene Expression and Phenotypic Markers in an NT2 Neurosphere Model of Neural Development

**DOI:** 10.1371/journal.pone.0058822

**Published:** 2013-03-19

**Authors:** Eric J. Hill, David A. Nagel, John D. O’Neil, Elizabeth Torr, Elizabeth K. Woehrling, Andrew Devitt, Michael D. Coleman

**Affiliations:** Aston Research Centre for Healthy Ageing, School of Life and Health Sciences, Aston University, Aston Triangle, Birmingham, United Kingdom; National Institutes of Health, United States of America

## Abstract

Mood stabilising drugs such as lithium (LiCl) and valproic acid (VPA) are the first line agents for treating conditions such as Bipolar disorder and Epilepsy. However, these drugs have potential developmental effects that are not fully understood. This study explores the use of a simple human neurosphere-based in vitro model to characterise the pharmacological and toxicological effects of LiCl and VPA using gene expression changes linked to phenotypic alterations in cells. Treatment with VPA and LiCl resulted in the differential expression of 331 and 164 genes respectively. In the subset of VPA targeted genes, 114 were downregulated whilst 217 genes were upregulated. In the subset of LiCl targeted genes, 73 were downregulated and 91 were upregulated. Gene ontology (GO) term enrichment analysis was used to highlight the most relevant GO terms associated with a given gene list following toxin exposure. In addition, in order to phenotypically anchor the gene expression data, changes in the heterogeneity of cell subtype populations and cell cycle phase were monitored using flow cytometry. Whilst LiCl exposure did not significantly alter the proportion of cells expressing markers for stem cells/undifferentiated cells (Oct4, SSEA4), neurons (Neurofilament M), astrocytes (GFAP) or cell cycle phase, the drug caused a 1.4-fold increase in total cell number. In contrast, exposure to VPA resulted in significant upregulation of Oct4, SSEA, Neurofilament M and GFAP with significant decreases in both G2/M phase cells and cell number. This neurosphere model might provide the basis of a human-based cellular approach for the regulatory exploration of developmental impact of potential toxic chemicals.

## Introduction

Valproic acid (VPA) and Lithium (LiCl) are commonly used drugs for the treatment of Bipolar disorder whilst VPA is also used in the treatment of various types of seizure. Although these agents have been used for decades, their pharmacological and toxicological mechanisms remain poorly understood. Of particular interest is their contribution to Developmental Neurotoxicity (DNT) as well as their potential impact on stem cells.

A number of in vitro model systems have been used to study DNT such as primary rodent cultures [1] as well as stem cell based models such as the mouse embryonic stem cell test [2], [3] and even mouse embryocarcinoma cells [4]. Human embryonic stem cells (hESc) can also be used to analyze the *in vitro* development from undifferentiated pluripotent cells leading to terminally differentiated cell types, recapitulating the process of early embryonic development [5]. Indeed, both the hESc and human neuroprogenitor cell (hNPC) models provide valid and useful tools for studying DNT. In addition, human models offer the advantage of better predictive power to man, since extrapolation of results across species is not an issue [6].

We have previously utilised an NT2.D1 neurosphere based model of neuronal differentiation to study the effects of a variety of compounds on neural development [7]. In this study we treated differentiating NT2.D1 neurospheres with LiCl or VPA in order to determine their effects using a toxicogenomic and phenotypic anchoring approach.

Toxicogenomics is a relevant approach for the identification of biomarkers associated with toxicity and can be employed in endpoint analysis following exposure to DNTs. To provide a sensitive and relevant endpoint, aberrations in gene regulation following exposure to potential teratogens should be linked to toxic outcomes, such as protein expression, cell proliferation and morphological changes. This is important, because changes in gene or protein expression alone may not be sufficient to differentiate toxicity from biological adaptation following exposure to a compound [8]. It may also be preferable to measure the significance of DNT effects on a group of genes from a pathway or functional category such as those defined in Gene Ontology (GO) terms because this facilitates the interpretation of the combined effects of gene changes and may markedly increase significance [9]. This grouping of genes also acknowledges that genes typically do not change in isolation and that it would be expected that any change that is causally related to toxicity would occur in a set of related genes rather than a single gene [10].

The objective of this study was to investigate the differential toxicity observed between LiCl and VPA [7] to further understand the developmental effects of these compounds. In this study we used full genome expression analysis combined with gene ontology (GO) analysis to identify key pathways. We then linked changes in gene expression to perturbations in differentiated cell populations and cell proliferation as phenotypic markers to functionally anchor the gene sets which were modified by toxicant exposure.

## Materials and Methods

### Materials

All chemicals were of molecular biology grade and were obtained from Sigma-Aldrich (Poole, UK) unless otherwise stated.

### NT2/D1 Cell Culture and Induction of Differentiation

NT2.D1 neurospheres were prepared as described [7]. Briefly human NT2.D1 cells were cultured in DMEM Glutamax high glucose medium, with pyruvate (Gibco Invitrogen, Paisley, UK) containing 10% v/v Heat inactivated foetal bovine serum (Gibco Invitrogen), 100 units/ml penicillin and 100 µg/ml streptomycin. Resuspended NT2/D1 cells (2×10^6^) were plated into sterile 90 mm diameter non-adherent bacteriological petri dishes (Starstedt, Leicester, UK) to generate neurospheres. These cultures were grown for 2 days before the addition of all-*trans*-retinoic acid (RA) to a final concentration of 10 µM together with either LiCl or VPA. RA only treated neurospheres were used as a control. Cells were replated and re-fed with RA containing media for the controls, or media containing RA and toxicants for the treated cells every 2–3 days. Suspension cultures were maintained for 2 weeks after which time neurospheres were used for analysis.

LiCl and VPA were diluted in sterile PBS to prepare a 100 mM stock solution and frozen at −20°C. Frozen aliquots were used only for each change of medium. Cells were treated with either 1 mM LiCl or 0.5 mM VPA. These concentrations have been previously shown to be within the range of patient plasma levels [11], [12]. In terms of fetal exposure to these drugs, the total concentration of VPA has been shown to be higher in cord serum than in the serum of epileptic mothers prescribed this drug [13], [14]. In comparison, lithium appears to equilibriate across the placenta over a range of maternal concentrations [15].

### RNA Extraction

Total RNA was extracted using Trizol reagent (Invitrogen) from 3 separate petri dishes and pooled for each replicate. RNA was treated with DNAase (Qiagen) for 30 minutes at room temperature. RNA was subsequently purified using the RNAeasy Kit (Qiagen). RNA quantification was performed using the Nanodrop 1000 (Thermofisher) and sample integrity was determined using an Agilent Bioanaylser.

### Two-Colour Microarray-based Gene Expression Analysis

Full genome analysis was carried out at Birmingham University Functional genomics laboratory (http://www.genomics.bham.ac.uk). Briefly, total RNA was extracted as outlined above. RNA samples were labelled using Agilent’s Quick AMP Labelling kit and hybridized to Agilent’s Whole human 44 K genome microarray. Slides were scanned using the Axon GenePix 4000B.

### Flow Cytometry

Flow cytometry was performed on disaggregated neurospheres following 14 days of differentiation. Neurospheres were washed three times with 10 ml PBS-0.1 M EDTA for 5 min. Washed neurospheres were pooled from 3 separate Petri dishes, centrifuged (217×G for 5 min) and resuspended in 5 ml Accutase (PAA laboratories, UK). Dissociated cells were washed twice with PBS, fixed in 4% formaldehyde (v/v in PBS) for 20 min on ice, washed twice in PBS-BS (PBS 0.2% BSA and 0.15% saponin) and incubated in PBS-BS containing 5% rabbit serum (RS) (Sigma Aldrich, UK) for 30 min on ice. The cells were then distributed between four microcentrifuge tubes and incubated with either mouse anti-NF-M (Millipore, UK) or anti-GFAP antibody (Millipore, UK), mouse anti-Oct4 antibody (Millipore, UK) and mouse anti-SSEA4 antibody (Abcam, UK) all diluted 1∶500 in PBS-BS with 5% RS for 1 h on ice. Cells were then washed three times with PBS-BS, and resuspended in rabbit anti-mouse Fluoroscein (FITC)-conjugated antibody (Jackson ImmunoResearch; diluted 1∶250 in PBS-BS with 5% RS) for 30 min on ice. The cells were washed three times and resuspended in PBS-BS. The percentage of fluorescent cells was then analysed using a Quanta SC flow cytometer (Beckman Coulter) gating on single cells only, with a total cell count of 10000 events. The background level was estimated by using samples in which the primary antibody was omitted.

### Cell Cycle Analysis

Neurospheres were harvested via centrifugation and washed 3 times in PBS 0.1 M EDTA then dissociated using Accutase (PAA) for 30 minutes at 37°C. Dissociated cells were washed 3 times in PBS 0.2% BSA, counted and resuspended in 1 ml PBS. Cells were fixed by the addition of 9 ml ice cold 70% ethanol and incubation at −20°C overnight. Subsequently fixed cells were centrifuged at 717×G and washed 3 times with PBS 0.1% BSA. Cells were then resuspended in 1 ml PBS containing 50 µg propidium iodide and 100 µg heat treated RNAse A and incubated for 3 hours in the dark at room temperature. Cells were analysed using a Quanta SC flow cytometer (Beckman Coulter) gating on single cells only with a total count of 10000 events.

### Protein Assay

Protein samples were prepared from NT2/D1 cells and their RA-induced derivatives in RIPA buffer (10 mM Tris HCL pH 8.0, 100 mM NaCl, 1 mM EDTA, 1% (v/v) NP40, 0.1% (w/v) SDS,0.5% (w/v) sodium deoxycholate) and one Complete mini EDTA-free protease inhibitor cocktail tablets/10 ml RIPA buffer (Roche Diagnostics Ltd). Protein concentration was determined using the BCA™ protein assay kit (Pierce, Rockford, USA).

### Gene Array

Data was normalised using the Lowess curve method, with RA as the control sample and VPA or LiCl as the treated sample (20% of the data was used to calculate the Lowess fit at each point). Probes were filtered by the criteria that in order to pass, the probes had to possess a Present or Marginal flag in at least 2 out of the 4 samples (4 samples, 2 groups with 2 replicates each). The remaining probes were used in the statistical filters for the 2 groups VPA and LiCl. A Student T-test applied to the Volcano plots was used to determine statistically significant differentially expressed genes from the filtered probes by comparing treatment with control (2 fold expression cutoff and a p-Value of 0.01).

### Realtime PCR

Five genes identified by Microarray analysis were selected for validation using SYBR green Realtime PCR. Total RNA was extracted as above. 1 µg of total RNA was reverse transcribed using Precision nanoscript™ reverse transcriptase (Primerdesign, Southampton UK) and oligo dT primers (PrimerDesign, Southampton, UK).

cDNAs were amplified in a standard 40-cycle SYBR® green real-time PCR reaction using optimised sequence specific pre-validated primers for Octamer 4 (Pou5F1), metallothionein (MT1G), mitogen-activated protein kinase 10 (MAPK10/JNK), NOTCH homologue 1 (NOTCH1) and Nestin (NES). Primers were supplied by PrimerDesign Ltd (Southampton UK), according to the manufacturer’s instructions.

GeNorm analysis (PrimerDesign Ltd, Southampton UK) was carried out to determine a housekeeping gene in which expression was not affected following treatment of cells. The house keeping genes Ubiquitin C (UBC), Eukaryotic initiation factor 4A2 (eIF4A2), 18S ribosomal RNA, Glyceraldehyde 3-Phosphate dehydrogenase (GAPDH), Beta-actin (ACTB), β2 Microglobulin (B2 M), Uncharacterised protein C14orf133 (VIPAR) and Glucose-6 phosphate dehydrogenase (G6PDH) were tested for stable expression following treatment of cells. Following analysis Ubiquitin C (UBC) was selected as a stably expressed gene for normalisation. The comparative CT method was used to calculate the relative quantification of gene expression. The following formula was used to calculate the relative amount of the transcripts in the chemical treated samples (treat) and the vehicle-treated samples (control), both of which were normalized to the endogenous controls. ΔΔCT = ΔCT (treat) – ΔCT (control) for biological RNA samples or ΔΔCT = ΔCT (HBRR) – ΔCT (UHRR) for reference RNA samples. ΔCT is the difference in CT between the target gene and endogenous controls by subtracting the average CT of controls from each replicate. The fold change for each treated sample (relative to the control sample (or UHRR)  = 2^–ΔΔCT^. Fold changes in gene expression using the comparative CT method and statistical analysis were determined using the freely available Relative Expression Software Tool (REST 2009, www.qiagen.com).

### Gene Ontology (GO) Studies

For pathway analysis of significantly modulated genes (two-tailed Student’s *t*-test, *p*-value <0.01) MetaCore™ (GeneGo, San Diego, USA) software was used. MetaCore™ is an online software suite that identifies and visualizes the involvement of differentially expressed genes in specific cellular pathways, which are subsequently related to the total amount of genes involved in the particular pathway and in all the available pathways combined (based upon the relative enrichment of genes and the saturation of each network with canonical pathways in Metacore GeneGo™). Enrichment analysis consists of matching gene IDs of possible targets for the "common", "similar" and "unique" sets with gene IDs in functional ontologies in MetaCore™. The probability of a random intersection between a set of IDs the size of target list with ontology entities is estimated in p-value of hypergeometric intersection. The ontologies include GeneGo Pathway Maps, GeneGo Process Networks, GO Processes, GeneGo Diseases (by Biomarkers). The degree of relevance to different categories for the uploaded datasets is defined by p-values, so that the lower p-value gets higher priority.

### Hierarchical Cluster Analysis

Unsupervised hierarchical clustering was performed on the differentially expressed genes common to both VPA and LiCl treatment using the online expression profiler described by Kapushesky *et al*, [16] using the following parameters: Euclidean distance using average linkage (average distance, UPGMA) and clustering rows.

### Statistical Analysis

Results were expressed as the mean of three samples ± standard error of the mean (SEM). Comparisons between treatments were performed using analysis of variance (ANOVA) with the appropriate post-test using GraphPad Prism Software. Differences were considered significant for p values <0.05.

## Results

### Microarray Analysis of NT2.D1 Neurosphere Gene Expression Following Exposure to LiCl and VPA

**Figure 1:**
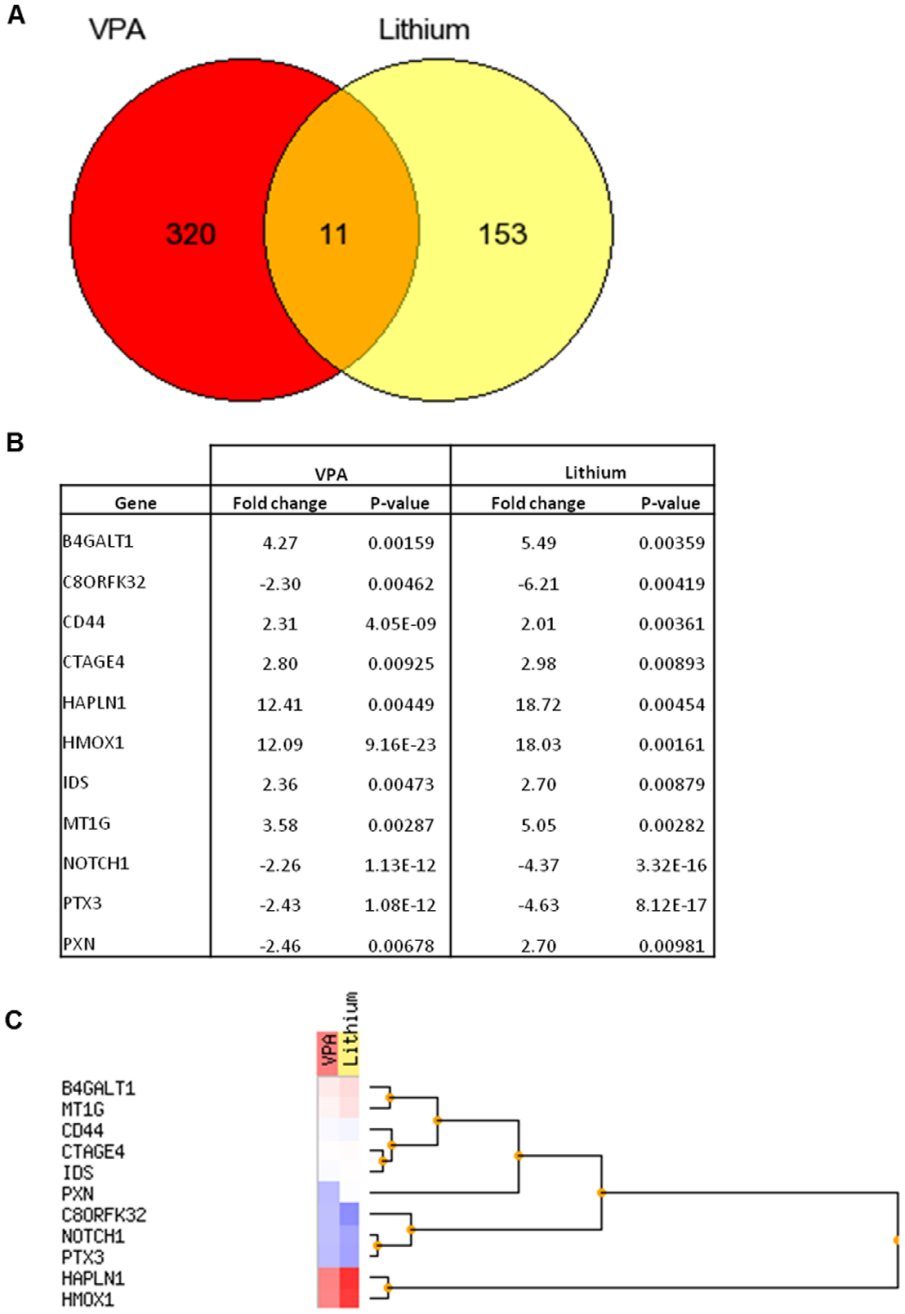
Venn diagram summarising the number of significantly differentially expressed genes unique and common to VPA+RA and LiCl+RA treatment (A). Tabulated fold change and p-value data (B) and unsupervised hierarchical cluster analysis (C) for the eleven genes found to be significantly up-regulated (red) or down-regulated (blue) in response to both to VPA and LiCl treatment.

RNA extracted from the NT2.D1 neurospheres treated with VPA or LiCl was subjected to microarray analysis using the Agilent whole genome 44K array. Following normalization the data was filtered to only include those genes differentially up or down-regulated by 2 fold or greater (Table S1, Table S2). Among the numerous genes (164) showing signs of deregulated expression following treatment with LiCl, 153 were unique (Fig. 1A). In the LiCl treated neurospheres 73 genes were downregulated and 91 were upregulated (Table S1). In comparison, amongst the numerous genes (331) showing signs of deregulated expression following treatment with VPA, 320 were unique (Fig. 1A), of those 114 were down-regulated and 217 genes were up-regulated by VPA (Table S2). Eleven genes were commonly deregulated by both VPA and LiCl (Fig. 1A and 1B). These eleven genes were split into groups by unsupervised hierarchical clustering analysis (Fig. 1C). Two main groups of up-regulated and down-regulated genes followed similar patterns in both the LiCl and VPA treated samples. A third group containing the paxillin (Pxn) encoding gene demonstrated an upregulation of the paxillin gene (2.70 fold) after exposure to LiCl with a similar magnitude down-regulation (2.46 fold) after exposure to VPA.

### Gene Ontology (GO) Analysis

GO analysis of the LiCl deregulated genes (Table 1) revealed a significant (p<0.05) overrepresentation of the following GO process networks; Signal transduction (NOTCH signalling), Cell cycle (G1-S Interleukin regulation), Cell cycle (G1-S Growth factor regulation). GO analysis of the VPA deregulated genes (Table 2) revealed a significant (p<0.05) overrepresentation of the following GO process networks; Cell adhesion (Cell junctions), Cell adhesion (Amyloid proteins), Development (Blood vessel morphogenesis), Development (Regulation of epithelial-to-mesenchymal transition), Proliferation (Negative regulation of cell proliferation), Cytoskeleton (Intermediate filaments) and Development (Neurogenesis:Synaptogenesis).

**Table 1:** Functional annotational clustering of genes enriched in NT2.D1 neurospheres following treatment with LiCl.

GO process network	Genes included in GO term	p value
Signal transduction_NOTCH signaling	CD44, DIP-1, ErbB3, JNK, Notch, p63, TGF-beta receptor type II	1.5E–4
Cell cycle_G1-S Interleukin regulation	Cyclin A, JNK, p14ARF, p15, p16INK4, STAT6	1.8E–4
Cell cycle_G1-S Growth factor regulation	Cyclin A, G-Protein alpha(i) family, JNK, p14ARF, P15, p16INK4, TGF-betareceptor type II	6.6E–4

Metacore™ (GeneGo) was used for this analysis using genes that were statistically significant p<0.05. Pathways with a p-value <0.05 were considered significantly modulated.

**Table 2:** Functional annotational clustering of genes enriched in NT2.D1 neurospheres following treatment with VPA.

GO process network	Genes included in GO term	p value
Cell adhesion_Cell junctions	Actin, Claudin-11, Claudin-7, desmoplakin, E-cadherin, EPB-41, keratin-18, keratin-8, Paxillin,PKC, Plakophilin-3, ZO-3	1.3E–05
Cell adhesion_Amyloid proteins	WNT, NOTCH, APLP2, G-alpha(q)specific frizzled GPCR, PKC, E-Cadherin, cortactin,X11-like, Actin	1.8E–05
Development_Blood vessel morphogenesis	Notch, G-alpha(q)specific frizzled GPCR, VHL, CEACAM1, HAND1, Pitx2, FOXC1/2, PDE,GRB14, SHP-2	4.1E–05
Development_EMT_Regulation of epithelial-to-mesenchymal transition	Notch, G-alpha(q)specific frizzled GPCR, Actin, Desmoplakin, E-cadherin, GSC, Keratin 18,keratin 8, MAP2K7, MMP-9, Nestin, Notch1, SHP-2, WNT	5.1E–05
Proliferation_Negative regulation of cell proliferation	BINPL, Calgizzarin, FRK, Galpha(i)-specific peptide GPCR, IBP, MAD3, MAD4, Pitx2, SHP-1,SSTR2, VHL	2.0E–04
Cytoskeleton_Intermediate filaments	Actin, desmoplakin, keratin 18, keratin 8, kinesis light chain, nestin	2.0E–3
Development_Neurogenesis:Synaptogenesis	Actin, Atrophin 1, Chapsyn-110, Cortactin, G-alpha(q)specific frizzled GPCR, N-type Ca(II)channel alpha1B, neuroligin, synaptotagmin, VAMP8, WNT, X11-like	2.0E–3
Cell adhesion_Cadherins	Actin, Cortactin, E-Cadherin, G-alpha(q)specific frizzled GPCR, k-cadherin, PKC, plakophilin,SHP-1, SHP-2, SWAP-70, WNT	2.0E–3
Development_Neurogenesis in general	FZD-5, G-alpha(q)specific frizzled GPCR, GFRalpha2, Nestin, Notch, PBX1, WNT	3.5E–3

Metacore™ (GeneGo) was used for this analysis using genes that were statistically significant p<0.05. Pathways with a p-value <0.05 were considered significantly modulated.

### Validation of Microarray Results Using Real-Time PCR

To assess the validity of altered expression of genes identified by Agilent’s Whole human 44K genome microarray analysis, we selected five genes for subsequent analysis by SYBR Green Real time PCR. Four genes that were changed in both gene sets upon analysis of the array data were included in addition to the gene Pou5F1, which was included to corroborate changes seen in the array data and the upregulation of expression of the protein product of this gene (Oct4) when analysed by flow cytometry. Genes included: Pou5F1, MT1G, MAPK10, NOTCH1 and NES. Genes expected to be upregulated by VPA and LiCl were significantly (p<0.05) induced (Table 3). Similarly, the genes expected to be downregulated by VPA and LiCl were significantly (p<0.05) decreased with the exception of NOTCH (p>0.05) (Table 3). Whilst a reduction in NOTCH1 expression was not found to be significant by Real Time PCR a reduction in expression was observed which was in agreement with the array data.

**Table 3:** Validation of microarray data using Real Time PCR.

	VPA			Lithium		
Gene	N-Fold	SEM (±)	p Value	N-Fold	SEM (±)	p Value
POU5F1	10.85	2.06	<0.05	1.81	0.13	<0.05
MT1G	15.30	3.58	<0.05	1.56	0.03	<0.05
MAPK10	−3.23	0.02	<0.05	−1.23	0.08	<0.05
NOTCH1	−2.20	0.04	>0.05	−1.70	0.03	>0.05
NES	−4.48	0.01	<0.05	1.04	0.14	<0.05

Data is expressed as fold expression changes of Pou5F1, MT1G, MAPK10, NOTCH1 and NES. Results are shown as fold change ±S.E.M (n = 3). Genes with a p-value <0.05 were considered significantly modulated.

### Proliferation of Cells Following Exposure to LiCl and VPA

**Figure 2:**
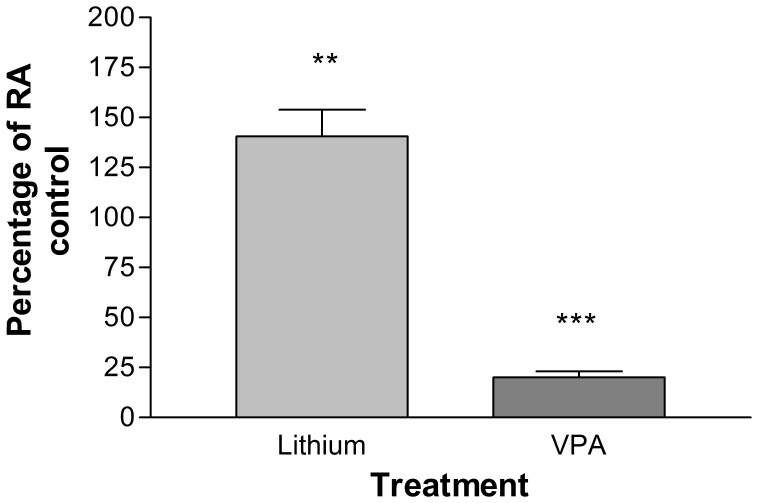
Total number of cells from dissociated neurospheres following treatment with VPA+RA and LiCl+RA as a percentage of the RA treated control. Cell populations of dissociated neurospheres post treatment with VPA (0.5 mM) and LiCl (1.0 mM). Results are shown as ± S.E.M (n = 3). p<0.05(*), p<0.01(**), p<0.001(***). p values were calculated by One-way ANOVA and corrected for multiple comparisons by Dunnets’s post-test in comparison to the RA only control.

Our previous data [7] did not demonstrate a significant increase in cell number using Cell Titre Blue following treatment of cells with LiCl at day 14 and only a modest <30% decrease in cell viability following treatment with VPA. However direct cell counting using a haemocytometer (Fig. 2) of dissociated neurospheres showed a significant increase (p<0.01) in the number of cells derived from neurospheres treated with 1 mM LiCl to 140.6±13.3% of RA treated control. In addition, there was a significant (p<0.001) decrease in the number of cells following treatment with VPA to 20±2.9% of the RA treated control. This data was also verified using flow cytometry (data not shown). Regarding the differences identified between direct cell counting and indirect measures such as cell viability used previously, we would now question the validity of using Cell Titre blue as a measure of cell number in 3D culture. To further validate the results of direct cell counting we also measured total protein and demonstrated almost identical results as that seen by direct cell counting (data not shown). This data would correlate with the changes in neurosphere size (increase with LiCl and a decrease with VPA) that were observed following exposure to toxins in both this and our previous study [7]. Whilst an increase in the number of cells could be correlated with just an increase in size, it is also possible that an increase in the number of neurospheres could also be responsible for the differences observed. As there is a wide distribution in the size and number of neurospheres, a measure of total cell numbers was found to be the most accurate method to determine cell proliferation.

### Cell Cycle Analysis of Cells Exposed to LiCl and VPA

**Figure 3:**
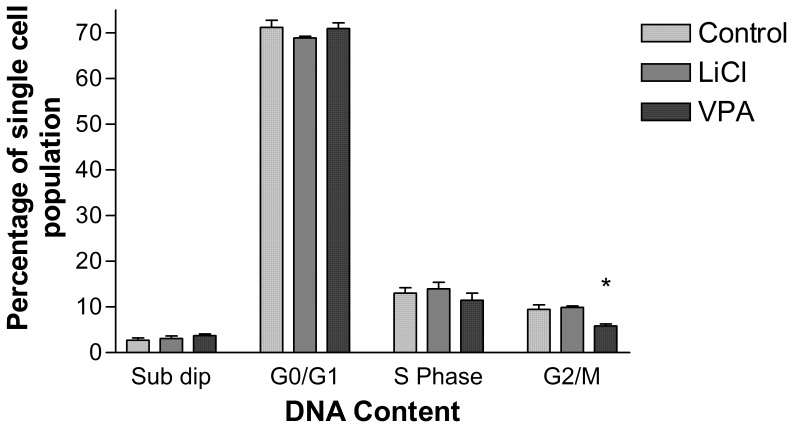
Cell cycle analysis of RA, LiCl+RA and VPA+RA treated cells. Cell populations of dissociated neurospheres in each cell cycle phase post treatment with VPA (0.5 mM) and LiCl (1.0 mM) and total cell number as a percentage of control cells post lithium and VPA treatment results are shown as ± S.E.M (n = 3). p<0.05(*), p<0.01(**), p<0.001(***). p values were calculated by One-way ANOVA and corrected for multiple comparisons by Bonferroni’s multiple comparison post-test in comparison to the RA only control.

Cell cycle analysis of cells exposed to LiCl using propidium iodide staining showed no significant changes compared to the control (Fig. 3). Conversely, cells treated with VPA showed a significant decrease (p<0.05) in the number of cells at the G2/M phase of the cell cycle.

### Flow Cytometry Analysis of Cells Treated with LiCl and VPA

**Figure 4:**
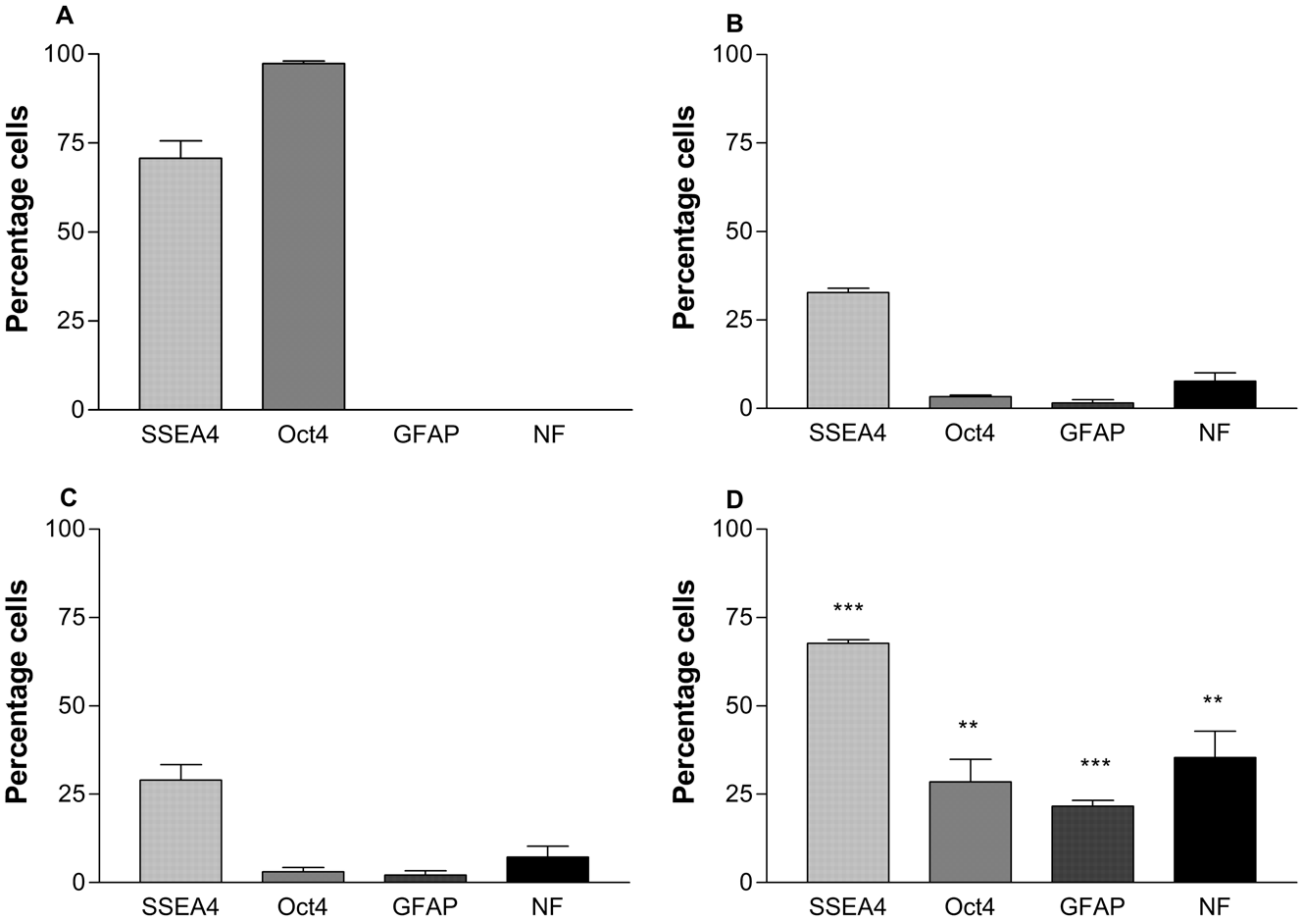
Proportion of cells expressing SSEA4, Oct4, GFAP and NFM following treatment with VPA+RA and LiCl+RA measured using flow cytometry. Number of cells expressed as a percentage of total number of cells A) Undifferentiated NT2.D1 cells, B) RA, C) LiCl (1 mM) and D) VPA (0.5 mM). Results are shown as ±S.E.M (n = 3). p<0.05(*), p<0.01(**), p<0.001(***). p values were calculated by One-way ANOVA and corrected for multiple comparisons by Bonferroni’s multiple comparison post-test in comparison to the RA only control.

Flow cytometry analysis of cells was used to determine the proportions of cells expressing stem cell associated markers (Oct4, SSEA4), a neuronal marker (NFM) and an astrocytic marker (GFAP) in both undifferentiated NT2.D1 cell, as well as RA and RA+toxicant treated neurospheres (Fig. 4). Undifferentiated NT2.D1 cells contain 70.7±4.9% and SSEA4, 97.3±0.7% Oct4 positive cells with no staining for GFAP or NFM. Differentiation with RA induces a significant reduction (p<0.001) in the levels of these pluripotentcy associated markers and an increase in the levels of cells expressing GFAP and NFM. Treatment with LiCl demonstrated no significant change in the proportions of cells expressing any of the markers tested from the RA treated control, despite a significant increase in the number of cells. In contrast, treatment with VPA resulted in a significant increase (p<0.001) in all of the markers tested compared to the RA treated control.

## Discussion

Well-characterized risks are associated with administration of VPA and LiCl during pregnancy. The rate for major congenital malformations for foetuses exposed to VPA is documented as approximately 6%–20% [17]. Whilst LiCl has been shown to disrupt development in diverse organisms, LiCl-related birth defects are rare in humans, but However, foetal exposure to LiCl in the first trimester may be associated with a small increased risk of Ebstein's anomaly [18]. Whilst LiCl has no reported developmental neurobehavioural effects, VPA-exposed children are three times more likely to require special education than controls [17].

In this preliminary study, the regulation of gene expression following chronic treatment with VPA and LiCl was explored in a neurosphere-based model of neurogenesis [7]. Phenotypic parameters including cell population changes, and cell cycle progression were also studied.

Cross-talk between cell signalling pathways is essential for measured and appropriate responses to stimuli and stress. This, therefore, requires the coordinated regulation of the expression of genes encoding integrated pathway components. Gene expression profiling therefore has the potential to reveal the molecular pathways that mediate the adverse responses to a toxicant. The initial output of toxicogenomics consists of a list of genes whose expression is altered upon toxicant exposure. In order to interpret this data in a biological context, bioinformatic methods such as GO mapping and pathway analysis can be used to complement gene expression profiling data [19].

In this study, gene expression analysis revealed only 11 common genes are shared by both LiCl and VPA. GO enrichment revealed that these shared genes are highly enriched in Wnt signalling through β-Catenin. This is perhaps unsurprising as VPA and LiCl inhibit the activity of glycogen synthase kinase 3 β (GSK3β). LiCl has been shown to be a direct, reversible inhibitor of GSK-3β with an IC_50_ value of ∼2 mM [20], as it acts through competitive inhibition of Mg^2+^. LiCl administered to mice has been shown to indirectly cause an increase in the phosphorylation of Ser9 of GSK-3β *in vivo.* As with LiCl, VPA has also been reported to inhibit GSK-3β enzymatic activity and induce GSK-3β^Ser9^ phosphorylation [21]. However, the finding that >90% of the genes altered by VPA and LiCl are unique would suggest that these two compounds exert their developmental actions by alternate mechanisms as evidenced by the overt phenotypic differences observed.

GO analysis using MetaCore™ (GeneGo) was used to identify enriched biological processes within differentially expressed genes. Biological processes such as signal transduction and cell cycle regulation were highlighted as the most significant GO categories following treatment with lithium. We also showed an increase in cell number following treatment with LiCl compared to the RA control. GO process network analysis of the LiCl treated cells also highlighted genes involved in cell cycle G1-S regulation which were affected by LiCl treatment. The increase in cell number associated with LiCl treatment would suggest cell cycle changes. However, whilst an increase in cell number was observed in this study, there were no significant changes in the cell cycle profile at 14 days differentiation. All cell treatments demonstrated a G0/G1 block which is one of the major effects of RA on multiple cell types [22] including EC cells [23] and appears to dominate the cell-cycle profile at day 14 of differentiation regardless of treatment. Future experiments will aim to determine the effects of LiCl on early cell cycle changes.

VPA exposure during the first trimester of pregnancy increases the risk for *spina bifida aperta*, a result of the failure of the posterior neural tube closure [24]. Several intracellular pathways, including the Ras-ERK pathway [25], have been shown to be affected by VPA, however, its major targets in the cells were found to be histone deacetylases (HDACs) [26],[27] and GSK3β [28]. The mechanism of teratogenicity is clearly distinct from that of the antiepileptic activity because VPA derivatives exist which are preferentially either teratogenic, such as *S*-4-yn-VPA, or antiepileptic, such as Valpromide (VPD). This is consistent with the proposition that disruption of proper embryonic development by VPA, but not the antiepileptic activity, is due to HDAC inhibition [29].

Treatment of neurospheres with VPA led to derangement of significantly more genes than LiCl. In addition, more overt phenotypic changes were also associated with this toxin in terms of cell number, protein expression and cell cycle stage. GO analysis was able to identify enriched biological processes within differentially expressed genes. Processes which are crucial during development such as cell adhesion, proliferation, cytoskeleton and development were highlighted as the most significant GO-categories.

In this study VPA treatment resulted in a significant decrease in cell number. VPA has been previously reported to regulate the differentiation and proliferation of neural progenitor and neuroblastoma cells [25],[30],[31]. It has been suggested that these effects are mediated by the ERK-p21^CIP/WAF1^ pathway [25].

Cell adhesion, cell polarity and cytoskeletal organisation play a fundamental role in driving morphogenesis in gastrulation and are essential in establishing the architecture of vertebrate embryos [32]. VPA has previously been shown to disrupt cell morphology and motility through interference with dynamics of the actin cytoskeleton [33]. Indeed, it has been shown that aberrations in these processes can result in neural tube defects [34]. The dynamics of adherens junctions and focal adhesions are controlled by the assembly and disassembly of adhesion components coupled with cytoskeletal remodelling actin organisation [35],[36]. In support of these findings, we observed changes in numerous genes associated with cell adhesion and the cytoskeleton in cells treated with VPA (Table 2). Previous studies using this system [7] identified significant changes in size and morphology of neurospheres which may have been linked with changes in cell adhesion and cytoskeletal organisation.

In accordance with the array data, flow cytometry indicated significant increases in the stem cell markers Oct4 and SSEA4 in the VPA treated neurospheres. Interestingly flow cytometric analysis revealed that the increase in the expression of the Pou5F1 gene seen in the array (13 fold) and PCR was correlated with an increase in the population of Oct4 expressing cells with a 5 fold increase in the number of cells positive for Oct4. It is has been shown previously that VPA specifically targets the proximal promoter region of Oct4 to activate its expression [37]. Indeed, VPA has been shown to improve reprogramming efficiency of induced pluripotent stem cells more than 100 fold using Oct4-GFP as a reporter [38]. The paradoxical expression of stem cell markers as well as differentiation markers such as GFAP and NFM could be explained by the nature of VPA in that it can directly drive the expression of Oct4 whilst concomitantly driving cell cycle exit and neural differentiation.

In summary, the gene changes highlighted in this study in combination with measurable phenotypic changes demonstrates the potential of combining expression profiling, GO analysis and phenotypic anchoring. We demonstrate that this approach may serve to enhance identification of biomarkers/pathways associated with the pharmacology and DNT of LiCL and VPA. Future studies will identify the temporal nature of these changes in order to fully understand the mechanism of action of these compounds. In addition, we will also determine the functional effects of these compounds on neurons and astrocytes derived from these cultures [39]. Following further validation with a larger range of toxins this approach may prove useful for identifying potential developmental neurotoxins as part of a battery of tests for regulatory screening processes.

## Supporting Information

Table S1 **Full list of genes differentially regulate by 1 mM LiCl in comparison to the RA treated control.** Expression changes with a p-value <0.05 were considered significantly modulated (n = 3).
(DOC)

Table S2 **Full list of genes differentially regulate by 0.5 mM VPA in comparison to the RA treated control.** Expression changes with a p-value <0.05 were considered significantly modulated (n = 3).
(DOC)
