# Supplementary material for: Effects of Lithium and Valproic Acid on Gene Expression and Phenotypic Markers in an NT2 Neurosphere Model of Neural Development
Source: PLoS One. 2013 Mar 19;8(3):e58822. doi: 10.1371/journal.pone.0058822 (PMC3602582; doi:10.1371/journal.pone.0058822)
Supplement: Table S1 — Full list of genes differentially regulate by 1 mM LiCl in comparison to the RA treated control. Expression changes with a p-value <0.05 were considered significantly modulated (n = 3). (DOC) [file pone.0058822.s001.doc]

| **Gene symbol** | **FC** | **p-value** |
| --- | --- | --- |
| ABHD11 | 2.93 | 0.0053 |
| ABI2 | -2.64 | 0.0037 |
| AF131834 | -2.89 | 0.0022 |
| AFG3L1 | -3.34 | 0.0049 |
| AK024315 | -2.01 | 0.0068 |
| AK055372 | -5.99 | 0.0097 |
| AK055386 | -2.35 | 0.0003 |
| ALOX5AP | 2.17 | 0.0084 |
| ALPPL2 | 7.99 | 0.0007 |
| ANXA11 | 2.70 | 0.0051 |
| APC2 | -4.83 | 0.0052 |
| APEH | 2.07 | 0.0006 |
| ASB1 | -2.04 | 0.0056 |
| B4GALT1 | 5.49 | 0.0036 |
| BC041417 | 2.84 | 0.0062 |
| BCL3 | 2.97 | 0.0034 |
| BST2 | 5.58 | 0.0067 |
| C19orf21 | 2.91 | 0.0024 |
| C1orf114 | -3.12 | 0.0083 |
| C6orf106 | 2.29 | 0.0085 |
| C8ORFK32 | -6.21 | 0.0042 |
| CARKL | 2.07 | 0.0039 |
| CASP4 | 5.42 | 0.0066 |
| CASP5 | 3.43 | 0.0065 |
| CCNA1 | -5.18 | 0.0073 |
| CD104030 | -2.35 | 0.0072 |
| CD44 | 2.01 | 0.0036 |
| CD97 | 2.62 | 0.0032 |
| CDC42EP2 | -2.54 | 0.0027 |
| CDC42SE1 | 2.01 | 0.0089 |
| CDC7 | -2.19 | 0.0078 |
| CDH2 | -2.00 | 0.0070 |
| CDKN2A | 15.67 | 0.0004 |
| CDKN2B | 13.45 | 0.0059 |
| CEBPD | 7.90 | 0.0027 |
| CENPF | -2.35 | 0.0020 |
| CEP250 | -3.38 | 0.0056 |
| CFD | 11.28 | 0.0059 |
| CFTR | 6.82 | 0.0000 |
| COL13A1 | -2.21 | 0.0049 |
| COVA1 | -2.17 | 0.0065 |
| CR597075 | -3.10 | 0.0091 |
| CR601260 | 2.06 | 0.0035 |
| CR601458 | 3.19 | 0.0006 |
| CR619250 | -2.67 | 0.0070 |
| CR626252 | 5.61 | 0.0061 |
| CTAGE4 | 2.98 | 0.0089 |
| CUGBP2 | -2.07 | 0.0022 |
| ENST00000292140 | 2.52 | 0.0047 |
| ENST00000308092 | -3.50 | 0.0060 |
| ENST00000312785 | 10.38 | 0.0075 |
| ENST00000334827 | -2.24 | 0.0056 |
| ERBB3 | 4.46 | 0.0000 |
| ESRRG | 2.66 | 0.0043 |
| F5 | 2.85 | 0.0000 |
| FAM104B | -2.17 | 0.0052 |
| FAM90A1 | -2.03 | 0.0058 |
| FLJ11286 | 5.31 | 0.0036 |
| FLJ20366 | 4.06 | 0.0052 |
| FLJ21963 | 4.19 | 0.0053 |
| FLJ33790 | -4.65 | 0.0004 |
| FLJ36032 | -3.48 | 0.0096 |
| FMN2 | -3.01 | 0.0093 |
| FXYD5 | 2.59 | 0.0099 |
| GBGT1 | 3.57 | 0.0099 |
| GDA | 3.89 | 0.0085 |
| GNAZ | -2.01 | 0.0012 |
| GNPAT | -2.23 | 0.0032 |
| GRAMD2 | 2.13 | 0.0040 |
| GRAMD3 | 4.02 | 0.0051 |
| GRN | 4.13 | 0.0047 |
| HAPLN1 | 18.72 | 0.0045 |
| HCP1 | 3.06 | 0.0009 |
| HKR2 | -2.05 | 0.0099 |
| HMOX1 | 18.03 | 0.0016 |
| HSPA12A | -2.77 | 0.0052 |
| HTR2A | -4.13 | 0.0075 |
| ICAM1 | 4.24 | 0.0006 |
| IDS | 2.70 | 0.0088 |
| IQCA | 2.37 | 0.0010 |
| JDP2 | 9.99 | 0.0087 |
| KBTBD11 | -2.59 | 0.0012 |
| KCNK6 | 3.07 | 0.0056 |
| KCNN4 | 7.82 | 0.0073 |
| KIAA0738 | -2.05 | 0.0012 |
| KIAA1143 | -2.17 | 0.0020 |
| KIAA1217 | 2.66 | 0.0030 |
| KIF5A | -2.19 | 0.0068 |
| KRT19 | 12.75 | 0.0057 |
| LAMC1 | 3.39 | 0.0041 |
| LCP1 | 18.20 | 0.0021 |
| LOC130074 | -2.13 | 0.0097 |
| LOC130576 | 6.99 | 0.0059 |
| LOC90246 | 7.58 | 0.0026 |
| MAGED2 | -2.01 | 0.0035 |
| MAPK10 | -3.24 | 0.0062 |
| MAPT | -2.48 | 0.0026 |
| MARVELD2 | 6.87 | 0.0086 |
| MIB1 | -2.23 | 0.0044 |
| MITF | 3.19 | 0.0084 |
| MLLT11 | -2.79 | 0.0068 |
| MMAB | -3.25 | 0.0098 |
| MT1G | 5.05 | 0.0028 |
| MYCT1 | 4.63 | 0.0084 |
| NHLH1 | -2.73 | 0.0090 |
| NOTCH1 | -4.37 | 0.0000 |
| NPEPL1 | 2.09 | 0.0078 |
| NTN1 | -2.76 | 0.0072 |
| OVOS2 | -2.32 | 0.0067 |
| P11 | 3.87 | 0.0051 |
| PDE4B | -2.72 | 0.0074 |
| PDGFRL | 3.72 | 0.0020 |
| PDIA5 | 2.08 | 0.0072 |
| PDXK | 2.80 | 0.0057 |
| PLAGL1 | -2.26 | 0.0096 |
| PLAU | 7.22 | 0.0000 |
| PLD1 | 6.95 | 0.0094 |
| PLEKHA6 | -2.81 | 0.0057 |
| POLR3B | -2.14 | 0.0031 |
| PPAP2C | 4.56 | 0.0008 |
| PPARG | 3.47 | 0.0000 |
| PPM1H | 3.07 | 0.0012 |
| PPP1R14C | -4.50 | 0.0100 |
| PSME1 | 2.09 | 0.0091 |
| PTX3 | -4.63 | 0.0000 |
| PURG | -2.14 | 0.0003 |
| PXN | 2.70 | 0.0098 |
| PYCARD | 6.95 | 0.0018 |
| RBM23 | -2.30 | 0.0028 |
| REXO2 | 2.57 | 0.0018 |
| RKHD3 | -2.35 | 0.0080 |
| RP11-262H14.4 | -2.56 | 0.0002 |
| RP11-262H14.4 | -2.29 | 0.0092 |
| RTN1 | -3.83 | 0.0030 |
| S100A3 | 3.77 | 0.0029 |
| SCNN1A | 5.96 | 0.0072 |
| SCPEP1 | 3.26 | 0.0052 |
| SHF | -2.32 | 0.0028 |
| SILV | 8.04 | 0.0053 |
| SNCA | 2.77 | 0.0000 |
| SPAG5 | -2.19 | 0.0094 |
| SQRDL | 3.40 | 0.0094 |
| STAT6 | 3.63 | 0.0035 |
| SUMF1 | 2.65 | 0.0005 |
| TERF2IP | -2.14 | 0.0084 |
| TGFBR2 | 3.56 | 0.0065 |
| THC2276324 | -2.00 | 0.0082 |
| THC2315854 | 2.24 | 0.0081 |
| THC2390143 | -2.08 | 0.0016 |
| THC2435791 | -2.00 | 0.0012 |
| TMPRSS2 | 4.07 | 0.0034 |
| TNNC1 | 3.11 | 0.0028 |
| TNNT1 | 9.38 | 0.0065 |
| TOP3A | -2.04 | 0.0079 |
| TP73L | 21.29 | 0.0006 |
| TPD52L1 | 10.69 | 0.0037 |
| TPM1 | 2.85 | 0.0077 |
| TRIB3 | 2.26 | 0.0059 |
| TRIM6 | 2.95 | 0.0021 |
| VIM | -3.61 | 0.0094 |
| WHSC1 | -2.50 | 0.0010 |
| YBX1 | -2.72 | 0.0083 |
| ZNF266 | -2.00 | 0.0041 |
| ZNF285 | -2.12 | 0.0061 |
| ZNF423 | -2.11 | 0.0090 |
