# Supplementary material for: Effects of Lithium and Valproic Acid on Gene Expression and Phenotypic Markers in an NT2 Neurosphere Model of Neural Development
Source: PLoS One. 2013 Mar 19;8(3):e58822. doi: 10.1371/journal.pone.0058822 (PMC3602582; doi:10.1371/journal.pone.0058822)
Supplement: Table S2 — Full list of genes differentially regulate by 0.5 mM VPA in comparison to the RA treated control. Expression changes with a p-value <0.05 were considered significantly modulated (n = 3). (DOC) [file pone.0058822.s002.doc]

| **Name** | **FC** | **p-value** |
| --- | --- | --- |
| ABCC2 | 2.66 | 0.0090 |
| ABP1 | 6.07 | 0.0088 |
| ACSL3 | 2.53 | 0.0018 |
| ACTA2 | -3.29 | 0.0064 |
| ADM | 5.73 | 0.0000 |
| AF339771 | 2.58 | 0.0074 |
| AGT | 2.03 | 0.0093 |
| AHNAK | 7.31 | 0.0097 |
| AI933337 | -3.17 | 0.0047 |
| AIM1L | 3.75 | 0.0046 |
| AJ276555 | 3.15 | 0.0006 |
| AK091784 | -3.60 | 0.0093 |
| AK123079 | 2.48 | 0.0020 |
| AKAP11 | -2.90 | 0.0021 |
| ANKK1 | -3.36 | 0.0086 |
| ANKRD13B | -2.74 | 0.0097 |
| ANKRD38 | 7.60 | 0.0028 |
| ANXA4 | 2.88 | 0.0096 |
| APBA2 | -2.28 | 0.0092 |
| APLP2 | 2.06 | 0.0028 |
| ARHGAP18 | 3.38 | 0.0016 |
| ARHGEF3 | 2.60 | 0.0009 |
| ATG5 | 2.13 | 0.0003 |
| ATN1 | -2.02 | 0.0035 |
| ATN1 | -3.60 | 0.0020 |
| ATP6AP2 | 2.54 | 0.0041 |
| ATP6V1C2 | 16.37 | 0.0094 |
| AVPI1 | 4.40 | 0.0065 |
| B2M | 2.94 | 0.0000 |
| B3GALT4 | 2.44 | 0.0091 |
| B4GALNT1 | -3.34 | 0.0064 |
| B4GALNT1 | -2.27 | 0.0032 |
| B4GALT1 | 4.27 | 0.0016 |
| BC030993 | -2.33 | 0.0071 |
| BC040420 | -4.57 | 0.0084 |
| BC073935 | 3.83 | 0.0094 |
| BCL2L14 | 3.74 | 0.0059 |
| BDP1 | -2.57 | 0.0081 |
| BG612665 | -2.15 | 0.0065 |
| BG777521 | 2.68 | 0.0098 |
| BHLHB2 | 2.29 | 0.0085 |
| BNIPL | 2.70 | 0.0080 |
| BRSK2 | -3.28 | 0.0078 |
| BRUNOL6 | -3.51 | 0.0027 |
| C10orf82 | 2.45 | 0.0005 |
| C11orf52 | 3.24 | 0.0025 |
| C15orf28 | -2.55 | 0.0072 |
| C15orf28 | -2.13 | 0.0003 |
| C20orf54 | 3.91 | 0.0027 |
| C21orf82 | -4.90 | 0.0005 |
| C2orf31 | 3.66 | 0.0075 |
| C3orf51 | -2.56 | 0.0039 |
| C8orf47 | 4.55 | 0.0014 |
| C8ORFK32 | -2.30 | 0.0046 |
| CA13 | 2.56 | 0.0054 |
| CCDC15 | -2.20 | 0.0088 |
| CCDC57 | -2.09 | 0.0005 |
| CCDC98 | 2.30 | 0.0000 |
| CCL26 | 10.46 | 0.0049 |
| CD177 | 2.06 | 0.0055 |
| CD302 | 3.18 | 0.0072 |
| CD44 | 2.31 | 0.0000 |
| CD55 | 4.15 | 0.0058 |
| CDCP1 | 4.22 | 0.0050 |
| CDH1 | 18.84 | 0.0004 |
| CEACAM1 | 3.68 | 0.0067 |
| CENTA2 | 3.02 | 0.0038 |
| CHMP2A | 2.17 | 0.0017 |
| CHMP4B | 2.15 | 0.0057 |
| CHST2 | 3.14 | 0.0036 |
| CLDN10 | 11.27 | 0.0003 |
| CLDN11 | -2.23 | 0.0008 |
| CLDN7 | 6.67 | 0.0037 |
| CLEC4D | 6.85 | 0.0016 |
| CPE | 2.23 | 0.0043 |
| CR618217 | -2.54 | 0.0050 |
| CSF1R | 3.72 | 0.0043 |
| CSF3R | 3.85 | 0.0014 |
| CTAGE4 | 2.80 | 0.0093 |
| CTSL | 2.78 | 0.0073 |
| CTTN | 2.08 | 0.0072 |
| CUTL2 | -2.02 | 0.0059 |
| CYP2S1 | 3.41 | 0.0033 |
| DARC | -2.29 | 0.0032 |
| DDEFL1 | -2.35 | 0.0011 |
| DDIT3 | 2.35 | 0.0098 |
| DDX58 | 2.24 | 0.0079 |
| DKFZp761E198 | -5.41 | 0.0008 |
| DLG2 | 2.35 | 0.0090 |
| DMKN | 4.96 | 0.0014 |
| DPF1 | -2.82 | 0.0009 |
| DPPA2 | 8.40 | 0.0049 |
| DSP | 2.30 | 0.0078 |
| DUSP26 | -2.09 | 0.0028 |
| DUSP5 | 5.14 | 0.0075 |
| E4F1 | -2.04 | 0.0058 |
| EFEMP1 | 5.96 | 0.0025 |
| EIF4E3 | 2.50 | 0.0097 |
| ELL2 | 3.62 | 0.0049 |
| ENC1 | -3.94 | 0.0100 |
| ENST00000233161 | 2.00 | 0.0073 |
| ENST00000258775 | -4.20 | 0.0096 |
| ENST00000288911 | -2.62 | 0.0036 |
| ENST00000295859 | 2.65 | 0.0086 |
| ENST00000321892 | 2.42 | 0.0097 |
| ENST00000326474 | -2.01 | 0.0039 |
| ENST00000327299 | 2.90 | 0.0022 |
| ENST00000371372 | -2.32 | 0.0086 |
| ENST00000373542 | 2.29 | 0.0037 |
| ENST00000375077 | 5.27 | 0.0018 |
| ENST00000375855 | 5.06 | 0.0064 |
| ENST00000376834 | 3.02 | 0.0034 |
| ENST00000379969 | 2.11 | 0.0058 |
| ENST00000382216 | -15.95 | 0.0067 |
| EPB41 | -4.24 | 0.0078 |
| ETV5 | 2.14 | 0.0041 |
| F2RL1 | 3.67 | 0.0093 |
| FAM38A | 2.42 | 0.0046 |
| FAM3C | 2.02 | 0.0011 |
| FILIP1 | 2.07 | 0.0058 |
| FKSG2 | 2.34 | 0.0057 |
| FLJ14327 | -2.18 | 0.0055 |
| FLJ22746 | 3.23 | 0.0045 |
| FLJ23152 | 2.43 | 0.0042 |
| FLJ25801 | 9.97 | 0.0058 |
| FLJ32658 | 4.53 | 0.0094 |
| FLJ36748 | 4.32 | 0.0054 |
| FLJ36840 | -3.37 | 0.0079 |
| FLJ40432 | 2.89 | 0.0072 |
| FLJ40432 | 3.32 | 0.0072 |
| FOXC1 | 4.02 | 0.0093 |
| FOXD2 | 2.37 | 0.0097 |
| FOXP4 | -2.87 | 0.0100 |
| FRK | 2.20 | 0.0008 |
| FTH1 | 3.25 | 0.0099 |
| FUT6 | -3.26 | 0.0096 |
| GAS5 | 2.18 | 0.0099 |
| GFRA2 | -3.73 | 0.0047 |
| GGTLA4 | 2.54 | 0.0062 |
| GM2A | 3.69 | 0.0043 |
| GNL3L | -4.33 | 0.0073 |
| GNPNAT1 | 2.23 | 0.0044 |
| GPR114 | 2.99 | 0.0059 |
| GPR126 | 2.88 | 0.0086 |
| GRB14 | 2.50 | 0.0072 |
| GRTP1 | 2.08 | 0.0065 |
| GSC | 2.72 | 0.0065 |
| GSH1 | -2.18 | 0.0070 |
| GSTO1 | 2.89 | 0.0017 |
| HAND1 | 3.16 | 0.0009 |
| HAPLN1 | 12.41 | 0.0045 |
| HIST1H2AH | -2.09 | 0.0018 |
| HIST2H2AC | -2.42 | 0.0069 |
| HIST3H2A | -2.24 | 0.0014 |
| HMOX1 | 12.09 | 0.0000 |
| HOOK1 | 2.97 | 0.0080 |
| HORMAD1 | 5.66 | 0.0020 |
| HSDL2 | -2.70 | 0.0052 |
| IDS | 2.36 | 0.0047 |
| IFIH1 | 4.76 | 0.0094 |
| IGFBP3 | 3.35 | 0.0000 |
| ITGB2 | 6.87 | 0.0000 |
| KDELR2 | 2.62 | 0.0008 |
| KIAA1804 | 2.30 | 0.0011 |
| KIAA1815 | 2.86 | 0.0087 |
| KIF13B | 2.64 | 0.0037 |
| KLC2 | -2.16 | 0.0010 |
| KLHDC8B | -3.65 | 0.0045 |
| KRT18 | 4.83 | 0.0031 |
| KRT8 | 3.17 | 0.0005 |
| LAD1 | 6.68 | 0.0040 |
| LAMP3 | 2.92 | 0.0029 |
| LFNG | -2.04 | 0.0005 |
| LIMD2 | -2.49 | 0.0088 |
| LIME1 | -2.19 | 0.0065 |
| LMAN1 | 4.06 | 0.0094 |
| LOC283454 | 5.51 | 0.0060 |
| LOC339047 | -3.83 | 0.0080 |
| LOC399851 | -2.15 | 0.0061 |
| LOC442075 | -2.48 | 0.0018 |
| LOC57228 | 5.90 | 0.0030 |
| LRRC49 | -2.02 | 0.0053 |
| LY6K | 5.13 | 0.0053 |
| MAN2A1 | 3.23 | 0.0012 |
| MAP2K7 | -2.51 | 0.0026 |
| MAST1 | -2.67 | 0.0004 |
| ME3 | -2.15 | 0.0051 |
| MERTK | 4.07 | 0.0013 |
| MGC33926 | -2.49 | 0.0007 |
| MIER3 | -4.41 | 0.0048 |
| MLPH | 2.79 | 0.0013 |
| MMP9 | 2.20 | 0.0045 |
| MT1A | 3.02 | 0.0029 |
| MT1B | 2.79 | 0.0091 |
| MT1F | 3.11 | 0.0080 |
| MT1G | 3.58 | 0.0029 |
| MT1H | 3.47 | 0.0085 |
| MUC16 | 2.81 | 0.0073 |
| MX1 | 4.93 | 0.0092 |
| MXD3 | -2.30 | 0.0018 |
| MXD4 | -2.46 | 0.0021 |
| MXRA8 | 2.05 | 0.0029 |
| MYO9B | -2.38 | 0.0093 |
| NDUFC1 | 2.22 | 0.0070 |
| NES | -4.57 | 0.0037 |
| NETO2 | -2.08 | 0.0085 |
| NFE2L3 | 10.67 | 0.0099 |
| NLGN2 | -2.70 | 0.0012 |
| NMI | 5.15 | 0.0000 |
| NOTCH1 | -2.26 | 0.0000 |
| NS3BP | -3.27 | 0.0029 |
| NUDT4 | 2.46 | 0.0068 |
| NUPR1 | 3.53 | 0.0081 |
| OAT | 3.51 | 0.0050 |
| OLFM1 | -2.00 | 0.0066 |
| OLFML3 | 4.05 | 0.0071 |
| PAQR4 | -2.56 | 0.0039 |
| PARP12 | 2.59 | 0.0020 |
| PBX1 | -2.16 | 0.0005 |
| PDE1A | -2.11 | 0.0065 |
| PDE2A | -2.17 | 0.0019 |
| PDE4C | -2.07 | 0.0027 |
| PDXP | -2.47 | 0.0012 |
| PDZD7 | -3.03 | 0.0091 |
| PERQ1 | -5.43 | 0.0025 |
| PGPEP1 | -3.13 | 0.0015 |
| PHF15 | 2.65 | 0.0067 |
| PHKA1 | 2.39 | 0.0065 |
| PITPNM1 | -2.15 | 0.0068 |
| PITX2 | 2.89 | 0.0093 |
| PKP3 | 7.20 | 0.0009 |
| PLA2G4A | 3.96 | 0.0000 |
| PLA2G7 | 2.78 | 0.0096 |
| PLB1 | 2.03 | 0.0054 |
| PLEKHF1 | 2.37 | 0.0013 |
| PLS3 | 2.38 | 0.0076 |
| POLE3 | -2.07 | 0.0052 |
| POU2F3 | 2.03 | 0.0068 |
| POU5F1 | 19.41 | 0.0009 |
| POU5F1 | 13.58 | 0.0081 |
| PPFIBP2 | 4.34 | 0.0027 |
| PPIC | 2.94 | 0.0013 |
| PPIC | 3.68 | 0.0029 |
| PPP1R12C | -2.94 | 0.0035 |
| PRKCZ | 2.05 | 0.0033 |
| PRNP | 2.36 | 0.0000 |
| PRODH | 3.82 | 0.0024 |
| PSMB9 | 11.37 | 0.0009 |
| PTPN11 | 2.00 | 0.0001 |
| PTPN6 | 2.49 | 0.0000 |
| PTPRD | -3.05 | 0.0021 |
| PTX3 | -2.43 | 0.0000 |
| PXN | -2.46 | 0.0068 |
| RAB25 | 8.15 | 0.0006 |
| RABEP2 | -2.62 | 0.0011 |
| RAG1 | 2.01 | 0.0091 |
| RAI1 | -2.38 | 0.0096 |
| RAP2C | 2.32 | 0.0097 |
| RBM35B | 8.01 | 0.0007 |
| RCOR1 | 2.37 | 0.0077 |
| RGC32 | 3.98 | 0.0088 |
| RGS12 | -2.26 | 0.0088 |
| RNF43 | 6.07 | 0.0023 |
| RP6-213H19.1 | 2.22 | 0.0079 |
| RPL22 | 2.34 | 0.0071 |
| RPL26 | 2.59 | 0.0037 |
| RPL26 | 2.39 | 0.0060 |
| RPL34 | 2.22 | 0.0065 |
| RPL5 | 2.34 | 0.0035 |
| RPS24 | 2.15 | 0.0065 |
| RUNX1T1 | -2.46 | 0.0004 |
| S100A11 | 4.91 | 0.0065 |
| SCGB3A2 | 3.61 | 0.0019 |
| SDC4 | 3.67 | 0.0019 |
| SELV | 2.61 | 0.0012 |
| SENP3 | -2.33 | 0.0090 |
| SH3D19 | 2.01 | 0.0044 |
| SH3RF1 | -2.07 | 0.0069 |
| SHD | -3.14 | 0.0060 |
| SIAH1 | -3.00 | 0.0069 |
| SLC16A4 | 4.39 | 0.0059 |
| SLC35D2 | 2.55 | 0.0020 |
| SLC35F2 | 3.81 | 0.0080 |
| SLC9A3R1 | 3.06 | 0.0088 |
| SMCR5 | 3.98 | 0.0077 |
| SMOC1 | 2.39 | 0.0049 |
| SPAG9 | -2.39 | 0.0032 |
| SPP1 | 6.60 | 0.0069 |
| SSTR2 | -2.45 | 0.0009 |
| SSX5 | -2.24 | 0.0020 |
| SUCLA2 | 2.11 | 0.0089 |
| SULT1C1 | 2.19 | 0.0032 |
| SWAP70 | 2.67 | 0.0007 |
| SYK | 3.86 | 0.0002 |
| SYNGR2 | 2.05 | 0.0004 |
| SYT12 | 2.06 | 0.0055 |
| TACSTD2 | 6.53 | 0.0068 |
| TAGLN3 | -2.37 | 0.0047 |
| TANC2 | -2.40 | 0.0079 |
| TBC1D16 | -2.19 | 0.0068 |
| TFRC | 2.46 | 0.0000 |
| THC2266397 | 3.22 | 0.0022 |
| THC2279115 | 5.24 | 0.0022 |
| THC2279735 | -2.01 | 0.0072 |
| THC2282954 | -2.33 | 0.0081 |
| THC2305336 | -5.18 | 0.0069 |
| THC2337941 | -3.18 | 0.0091 |
| THC2360930 | 2.06 | 0.0080 |
| THC2372472 | 3.86 | 0.0046 |
| THC2404058 | -3.73 | 0.0089 |
| THC2435579 | 2.96 | 0.0008 |
| THC2436337 | 3.06 | 0.0047 |
| THC2455550 | 2.28 | 0.0026 |
| TJP3 | 3.12 | 0.0040 |
| TMEM142A | -2.72 | 0.0035 |
| TMEM77 | 2.11 | 0.0066 |
| TMEM92 | 2.21 | 0.0088 |
| TPST2 | 3.12 | 0.0005 |
| TRIM63 | 6.83 | 0.0073 |
| UBE2G2 | -2.18 | 0.0003 |
| UBE2H | 2.58 | 0.0060 |
| USP6 | 2.10 | 0.0057 |
| VAMP8 | 7.73 | 0.0032 |
| VHL | -2.38 | 0.0066 |
| VIL2 | 3.70 | 0.0060 |
| WBSCR19 | -2.62 | 0.0038 |
| WDR63 | 2.20 | 0.0006 |
| WNT2B | -2.19 | 0.0019 |
| YPEL2 | 4.15 | 0.0014 |
| ZADH1 | 2.91 | 0.0021 |
| ZFHX4 | -2.35 | 0.0046 |
| ZKSCAN1 | -2.26 | 0.0001 |
| ZNF217 | 3.75 | 0.0099 |
| ZNF395 | 2.35 | 0.0069 |
| ZNF488 | -2.78 | 0.0080 |
| ZNF600 | 4.76 | 0.0005 |
| ZNF664 | -7.94 | 0.0053 |
